# Development of patient-specific iPSC-based epilepsy models and identification of differentially expressed genes for disease mechanisms

**DOI:** 10.3389/fnins.2025.1582255

**Published:** 2025-06-17

**Authors:** Jianfeng Wu, Siqi Huang, Lihao Chen, Yinghong Yang, Shunhan Zhang, Jiajia Xian, Xiaoyan Ma, Furong Ma, Shaoying Li, Yi Yang, Yingjun Xie, Xiaofang Sun

**Affiliations:** ^1^Department of Obstetrics and Gynecology, Guangdong Provincial Key Laboratory of Major Obstetric Diseases, Guangdong Provincial Clinical Research Center for Obstetrics and Gynecology, Guangdong-Hong Kong-Macao Greater Bay Area Higher Education Joint Laboratory of Maternal-Fetal Medicine, The Third Affiliated Hospital, Guangzhou Medical University, Guangzhou, China; ^2^Department of Obstetrics and Gynecology, Experimental Department of Obstetrics and Gynecology Institute, The Third Affiliated Hospital of Guangzhou Medical University, Guangzhou, China; ^3^Department of Clinical Laboratory Medicine, Guangdong Provincial Key Laboratory of Major Obstetric Diseases, Guangdong Center for Provincial Clinical Research Obstetrics and Gynecology, The Third Affiliated Hospital, Guangzhou Medical University, Guangzhou, China

**Keywords:** epilepsy, induced pluripotent stem cells (iPSCs), RNA sequencing, disease modeling, personalized medicine, gene expression analysis

## Abstract

**Introduction:**

Epilepsy is a complex neurodegenerative disorder characterized by recurrent seizures, which poses significant challenges for clinical management and therapeutic development. Recent advances in stem cell biology have enabled the use of patient-specific induced pluripotent stem cells (iPSCs) as a novel in vitro model for studying human diseases, including neurological disorders. In particular, iPSCs offer a promising platform for investigating the molecular mechanisms of epilepsy and facilitating personalized medicine.

**Methods:**

In this study, we generated iPSC lines from individuals diagnosed with epilepsy who carry a novel mutation in the *CLCNKB* gene. These patient-derived iPSCs were reprogrammed from somatic cells and subsequently characterized. To explore the molecular alterations associated with epilepsy, we performed transcriptomic profiling using RNA sequencing (RNA-seq). Differential gene expression analysis was conducted to compare the patient-derived iPSCs with control iPSC lines.

**Results:**

The RNA-seq analysis revealed a set of differentially expressed genes in epilepsy-derived iPSCs, with both upregulated and downregulated genes identified relative to controls. Several of these genes have previously been implicated in epilepsy-related phenotypes, as supported by published literature. This suggests a potential role for these genes in the pathophysiology of epilepsy linked to *CLCNKB* mutations.

**Discussion:**

The successful establishment of *CLCNKB*-mutant patient-specific iPSC lines and their transcriptomic characterization provide a valuable tool for studying the molecular basis of epilepsy. These findings underscore the potential of iPSC-based models to advance our understanding of disease mechanisms and support the development of novel diagnostic and therapeutic strategies. Moreover, this study demonstrates the applicability of iPSCs for epilepsy research and their promise in the field of personalized medicine.

## Graphical abstract

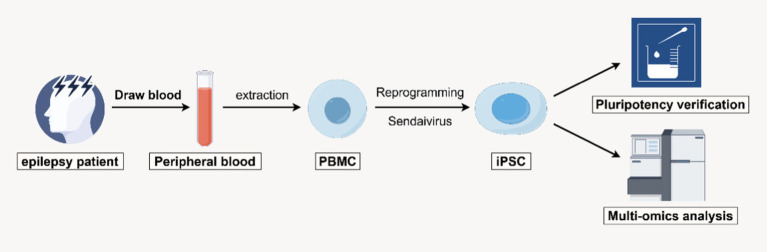

## Introduction

Epilepsy, a chronic neurological disorder characterized by recurrent, unprovoked seizures, is a significant public health concern affecting millions of individuals worldwide. Historically, the definition of epilepsy has been somewhat vague. A typical group of seizure symptoms has been grouped under the umbrella of epilepsy; however, not all seizures are epileptic, such as febrile seizures or drug - induced seizures (Thijs et al., 2019; Wirrell et al., 2022). Epileptic disorders are characterized by spontaneous seizures that can be long - lasting, frequent, or short - term, and they are accompanied by a number of neurobiological, cognitive, and psychosocial consequences (Singh and Sander, 2020). Although epilepsy is broadly defined, the clinical diagnosis should be as specific and precise as possible (Symonds et al., 2017). A key challenge in this field is to decipher the molecular mechanisms by which specific genetic variants disrupt neuronal excitability, as this knowledge is critical for the development of targeted therapies. Among the genes implicated in hereditary epilepsy, *CLCNKB* is a key player due to its role in regulating chloride homeostasis, an important process in maintaining the balance between neuronal membrane potential and excitability.

*CLCNKB* is a chloride ion channel gene that is expressed primarily in the kidneys and nervous system (Zhou et al., 2023; Nozu et al., 2008). It plays an essential role in maintaining ion balance both inside and outside the cells, particularly in regulating neuronal excitability. Previous studies have demonstrated that mutations in *CLCNKB* are closely associated with chloride channel dysfunction. This dysfunction, in turn, affects the neuronal membrane potential, leading to excessive neuronal excitability and epileptic seizures. For instance, mutations in the *CLCNKB* gene have been identified in several hereditary epilepsy syndromes, especially those associated with recurrent forms of epilepsy (Ferraguti et al., 2023).

In contrast, *LARGE1* is a gene associated with glycosylation and glycan modifications and is expressed primarily in the nervous system and other tissues (Beltrán et al., 2019). Recent studies have shown that glycan modifications play important roles in neural development, synaptic transmission, and the stability of neural networks. Abnormal glycosylation can lead to various neurodevelopmental disorders, including epilepsy (Righino et al., 2020). Specifically, *LARGE1* plays a crucial role in modifying the glycan structures on the surface of neural cells, and these alterations may affect synaptic plasticity and signal transmission between neurons, thereby promoting the development of epilepsy (Finsterer, 2019). Roos et al. (2024) found that *LARGE1* is dysregulated in spinal muscular atrophy (SMA) patients, leading to severe muscle atrophy. Clement et al. reported that the brain is involved in muscular dystrophy associated with defects in the glycosylation of dystroglycan caused by *LARGE1* (Clement et al., 2008). Additionally, Inamori et al. (2014) found that mutations in *LARGE1* are associated with abnormal brain development, underscoring its importance in neurodevelopment. While direct studies linking *LARGE1* to epilepsy are limited, existing evidence suggests that its role in neurodevelopment should not be overlooked, especially given the increasing attention given to the relationship between glycosylation and neurological diseases.

Traditional animal models possess inherent limitations when it comes to recapitulating the intricacies of human epilepsy and are often unable to accurately mirror the genetic and molecular heterogeneity witnessed in epilepsy patients. Induced pluripotent stem cells (iPSCs) have emerged as a valuable tool in disease modeling, as they offer the potential to generate patient-specific cells that can recapitulate the molecular and cellular features of human diseases (Hirose et al., 2020). iPSCs derived from individuals with epilepsy have the potential to recapitulate the molecular and cellular features of the disease, providing a unique platform for studying disease mechanisms and testing novel therapeutic approaches. The use of iPSCs to model epilepsy has gained considerable attention because of their ability to preserve genetic and epigenetic information from the patient’s original cells. This approach allows researchers to investigate the onset and progression of epilepsy in a context that closely mirrors the human condition. Moreover, iPSCs can be differentiated into various cell types, including neurons, glia, and astrocytes, which are central to the pathophysiology of epilepsy.

In this study, we aimed to establish patient-specific induced iPSC lines from individuals with epilepsy and to characterize their transcriptomic profiles using RNA sequencing (RNA-seq). Initially, we identified a mutation in the *CLCNKB* gene in the proband. However, since *CLCNKB* has not been previously reported to be associated with epilepsy, we expanded our analysis to include RNA-seq-based transcriptomic profiling. Through this broader approach, we sought to uncover other potential genetic variations or dysregulated pathways that may underlie the disease phenotype observed in this family. By comparing the gene expression patterns of these iPSC-derived cells to those of control cells, we sought to identify differentially expressed genes that may be associated with epilepsy pathophysiology. In our study, *CLCNKB* was identified through genetic screening in an epilepsy-affected family, whereas *LARGE1* emerged from subsequent sequencing data analysis. Based on their potential interaction and co-expression patterns, we hypothesize that both genes may contribute to the pathogenesis of epilepsy, which we aim to explore using patient-derived iPSC models. This study not only contributes to the development of a robust epilepsy disease model but also provides insights into the molecular mechanisms underlying epilepsy and opens new avenues for personalized medicine in epilepsy research. The results of this study are expected to advance our understanding of epilepsy and facilitate the development of novel diagnostic and therapeutic strategies tailored to individual patients.

## Materials and methods

### Source of clinical samples

Ethical approval for this study was obtained from the Academic Committee of the Third Affiliated Hospital of Guangzhou Medical University (Guangzhou, China; Approval No. YLKS2024-193). The samples used in this study were obtained from clinical epilepsy samples at the Third Affiliated Hospital of Guangzhou Medical University. The family under investigation included parents and their son, who was a 38-year-old epilepsy patient. The proband, demonstrated classic features of epilepsy starting at approximately 3 years of age. The patient exhibited involuntary hand movements characterized by muscle rigidity and clonic seizures. One hand showed significant weakness, resulting in difficulty performing coordinated movements and a marked impairment in motor function. The proband experienced slow and effortful movement, reflecting overall motor impairment. These clinical symptoms were recurrent and unprovoked, significantly impacting daily activities and motor control. The onset of epilepsy occurred at approximately the age of 3, with clinical manifestations including sudden loss of consciousness, generalized seizures, limited movement in the right hand, and difficulty walking. A report from the Second Affiliated Hospital of Guangzhou Medical University revealed a heterozygous nucleotide variation (c.228C > A) in the *CLCNKB* gene of the patient through whole - genome sequencing based on the detection of peripheral blood samples. The father carries the same mutation but does not exhibit any clinical symptoms. After informed consent was obtained from the patient, we collected peripheral blood and extracted peripheral blood mononuclear cells for subsequent experiments.

### PBMC extraction and experimental grouping

We extracted 15 mL of peripheral blood from each family member, including the father, mother, and son. Peripheral blood mononuclear cells (PBMCs) were isolated using Lymphoprep™ density gradient centrifugation. The resulting PBMCs were resuspended in cryopreservation solution at a concentration of 2 × 10^6^ cells/ml per tube and stored in liquid nitrogen for subsequent experiments, including transfection procedures.

In this family, the mother is clinically healthy with no known genetic disorders and serves as the control subject. She was selected as a control because of her lack of epilepsy phenotypes and the shared genetic background with the proband. In contrast, the father is a heterozygous carrier of the target gene (*CLCNKB*) mutation and is suspected to be affected by other potential genetic conditions. The son, who presents with clinical symptoms, shares the same pathogenic gene of interest. Therefore, the father and son are categorized as the experimental group in this study.

Statement: Reprogramming to induced pluripotent stem cells (iPSCs) eliminates age - associated transcriptomic differences (Lo Sardo et al., 2017; Singh and Newman, 2018).

### Reprogramming PBMCs into iPSCs

PBMCs were cultured in complete PBMC medium for 4 days, and PBMCs were transfected with Sendaivirus [CytoTune-iPS 2.0 Sendai Reprogramming Kit (Life Invitrogen, A16517)] on the fourth day. The transfected cells were cultured at 37°C and 5% CO_2_. After the growth of individual colonies, the monoclonal iPSCs were cultured in induction medium for several days, and the monoclonal iPSCs were transferred to mTeSR1/Matrigel culture system at 37°C and 5% CO_2_.

### Karyotyping

Karyotype analysis was conducted using iPSCs cultured to passage 10 using conventional G-band technology. At least 25 mid-term readings (550-band resolution) were performed for each sample.

Alkaline phosphatase staining (AP Staining): cultured iPSCs were fixed for 20 min with 4% paraformaldehyde (PFA) (Sigma, 30,525-89-4) and incubated with AP staining solution (Beyotime, C3206). The stained cells were observed under a microscope, and pictures were taken.

### Assay for STR

A genome kit was used to extract the patient’s blood as well as the genome of the iPSCs for STR testing.

### Immunofluorescence staining

When the cell density reached 50–60%, 4% paraformaldehyde and 0.5% Triton-X-100 were added for fixation and permeabilization, respectively. The corresponding primary antibody was added to each well after blocking with 2% BSA, and then the cells were incubated overnight at 4°C. The primary antibodies were OCT4 (abcam, 1:200), SOX2 (CST, 1:300), NANOG (CST,1:400), and SSEA4 (abcam,1:250), and the antibody parameters were as follows. The primary antibody was removed, a fluorescent secondary antibody was added, and the sections were incubated in the dark for 2 h. The samples were washed again and then 1 μg/mL DAPI was added for 10 min. Finally, the cells were washed several times with an appropriate amount of PBS and the samples were observed and imaged via a laser confocal microscope.

### Teratoma experiment

iPSCs (5 × 10^6^) were resuspended in mTeSR™ Plus medium supplemented with 50% Matrigel and injected subcutaneously into the groin of 4-week-old NOD/SCID mice. After 6–8 weeks, the material was removed and stained with hematoxylin and eosin for photographic observation.

### Flow cytometry analysis of the efficiency of cell pluripotency

iPSCs were cultured for 3–4 days, washed twice with D-PBS, digested with Accutase for 3 min, aborted with mTeSR™ Plus, and discarded by centrifugation. The cells were resuspended in 2% FBS-PBS and divided equally into 4 tubes for flow cytometry. The samples were permeabilized and fixed with BD Cytofix/Cytoperm™ for 15 min at room temperature and washed twice with BD Perm/Wash™ buffer. Then, the diluted primary antibodies (OCT4, SOX2, SSEA4, NANOG) were added and the mixture was incubated at room temperature for 1 h and washed twice with BD Cytofix/Cytoperm™. The corresponding secondary antibodies were subsequently added, the mixture was incubated at room temperature in the dark for 25 min, the mixture was washed twice, D-PBS was added, and the mixture was resuspended and detected on a machine.

### RNA-seq, gene ontology (GO) and kyoto encyclopedia of genes and experimental method for constructing the protein–protein interaction network

RNA was extracted from three independent induced pluripotent stem cell (iPSC) lines per group (biological replicates; *n* = 3 iPSC clones/group). Subsequently, the extracted RNA was subjected to high - throughput RNA sequencing (RNA - seq) with sufficient sequencing depth to ensure technical reproducibility.

Differential gene expression analysis was carried out based on the RNA - seq data obtained from these three biologically independent iPSC clones. This analysis was performed using the DESeq2 package, which utilizes a negative binomial distribution model to estimate gene - wise dispersion and calculate p - values for the identification of differentially expressed genes.

The identified differentially expressed genes were further analyzed and functionally predicted using the Gene Ontology (GO) database.^1^ To minimize the interference from a large number of non - informative genes among the differentially expressed genes, we applied a more stringent filtering criterion. Specifically, we increased the fold change threshold to 5 (FC > 5) while maintaining a significance level of *p* < 0.05. Moreover, genes with low expression levels (counts per million, CPM < 1) were excluded during data preprocessing to enhance the reliability of the results.

**Figure 1:**
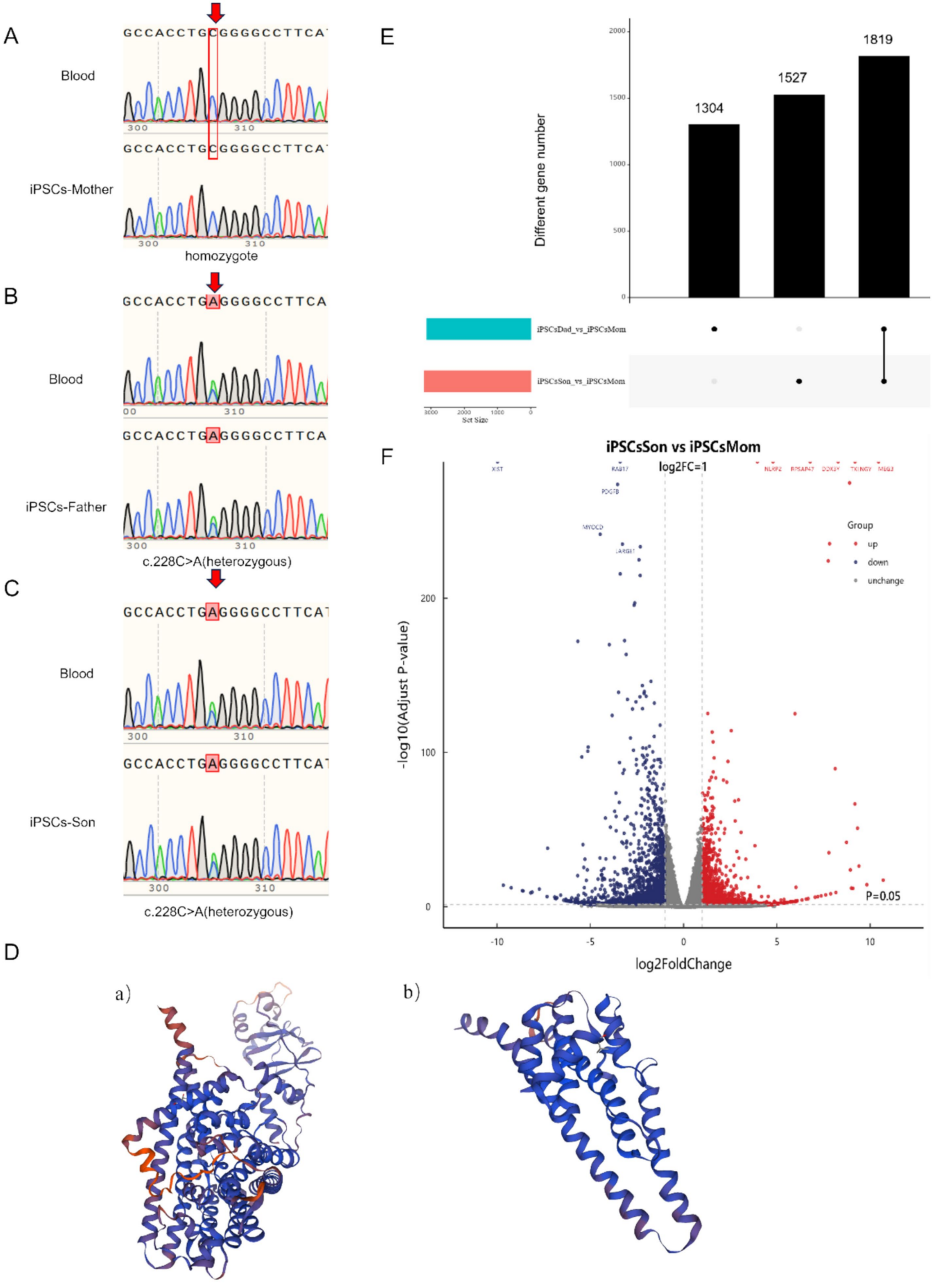
**(A–C)** Gene comparison before and after cell line establishment. (Peak overlaps (arrows) indicate heterozygosity at c.228C > A locus) **(D) (a)** Protein structure of the normal *CLCNKB* gene. **(D)** **(b)** Predicted protein structure of the mutated *CLCNKB* gene. **(E)** Number of differential genes in the patient’s family. **(F)** Volcano Plot depicting differentially expressed genes in the patient’s family, with Red Dots representing upregulated genes and Blue Dots representing downregulated genes.

As a consequence of this strict filtering and false discovery rate (FDR) correction, some genes in Figure 1F display extreme log₂ fold change (log₂FC) values. Based on these criteria, we selected the gene with the highest fold change for in - depth analysis.

## Results

### Establishment of monoclonal iPSCs and verification of pluripotency

**Figure 2:**
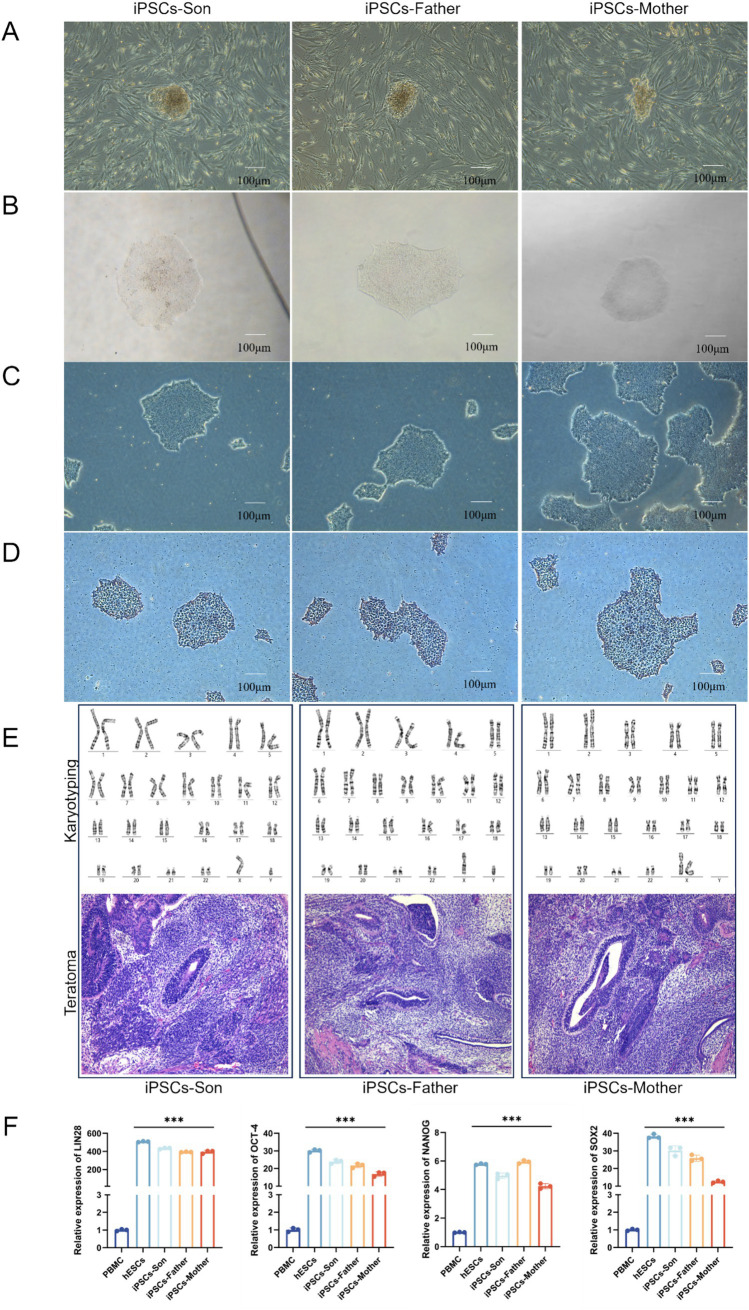
**(A)** Emergence of iPSCs during reprogramming. **(B)** Emergence of monoclonal cells after iPSC purification. **(C)** iPSCs after splitting and proliferating. **(D)** Alkaline phosphatase-stained iPSCs. **(E)** Karyotype analysis of iPSCs after successful induction and Histological analysis of teratomas formed from iPSCs. **(F)** The mRNA levels of LIN28, NANOG, OCT4, and SOX2 in iPSCs were detected via RT–qPCR (***p < 0.001).

We first used Sendai virus to generate the iPSCs shown in Figure 2. The presence of iPSCs during reprogramming and the selection of cells containing feeders (“Feeder” refers to feeder cells that support the growth and maintenance of pluripotent stem cells; in our study, mouse embryonic fibroblasts (MEFs) were used to maintain the undifferentiated state of iPSCs) for growth were verified (Figure 2A). To verify whether the iPSC chromosomes are abnormal, karyotype analysis was performed, which revealed no chromosomal abnormalities. Additionally, the results showed that when the cells were transplanted into immunodeficient mice, they formed teratomas containing tissues derived from all three germ layers—ectoderm, mesoderm, and endoderm (Figure 2E). The mRNA expression of the stem cell pluripotency indices LIN28, NANOG, OCT4 and SOX2 was detected via RT–PCR. Compared with those of the PBMCs, the mRNA expression levels of the newly established iPSCs were significantly greater (*p* < 0.001) (Figure 2F).

### Flow cytometry and immunofluorescence demonstrated the pluripotency of iPSCs

**Figure 3:**
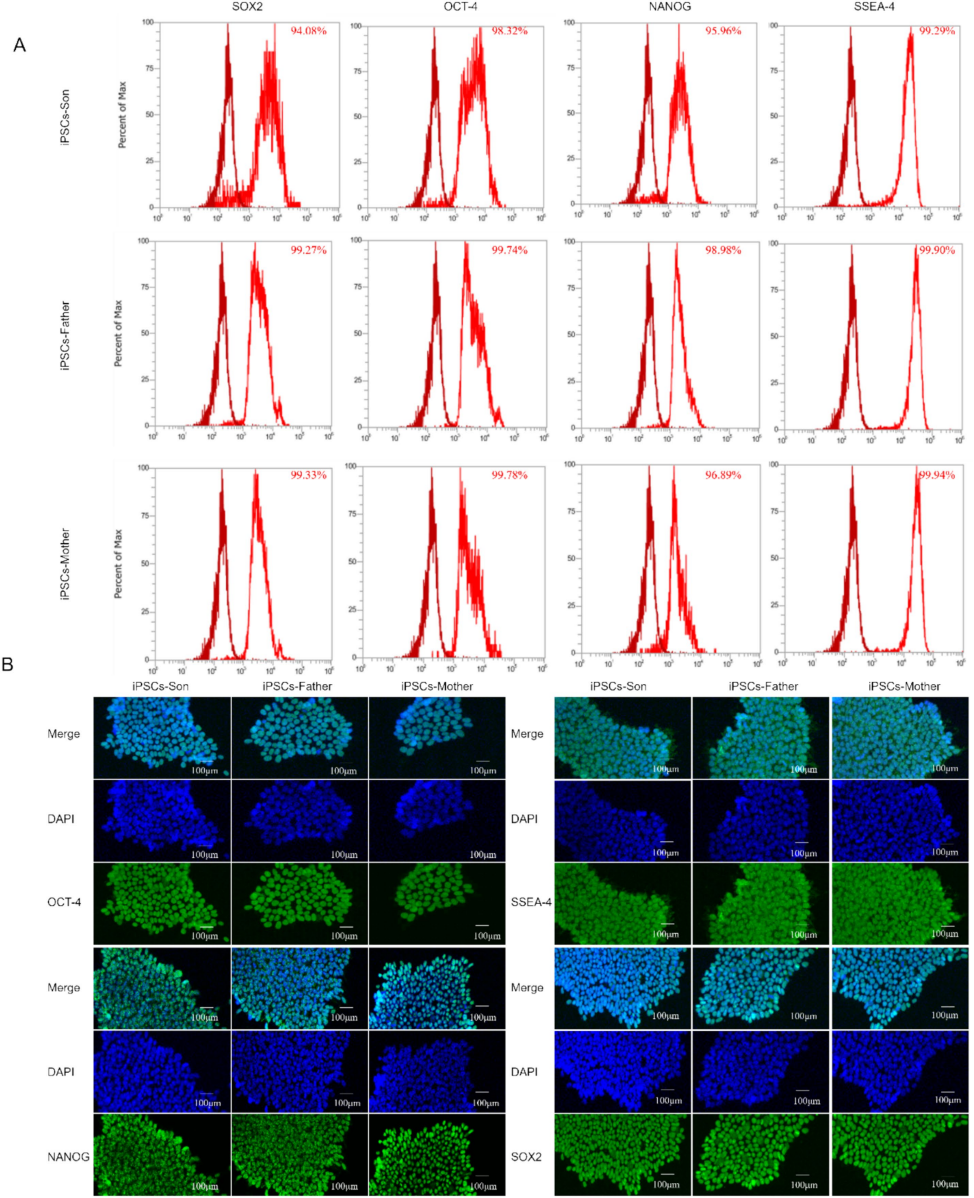
**(A)** Flow cytometry was used to analyse the pluripotency of iPSCs. **(B)** Immunofluorescence analysis of the iPSC cell markers *OCT-4, NANOG, SSEA-4,* and *SOX2.*

We used flow cytometry to detect the expression of the pluripotency markers OCT4, SOX2, NANOG, and SSEA4 in cells. As shown in Figure 3A, OCT4, SOX2, NANOG, and SSEA4 were highly expressed in cells. After the iPSCs were cultured in the mTeSR1/Matrigel system, their pluripotency was detected by immunofluorescence analysis. Pluripotency of the iPSC line was confirmed by robust expression of OCT4, SOX2, NANOG, and SSEA4 via immunofluorescence (Figure 3B). “robust expression” was defined as >90% of cells showing nuclear (OCT4, SOX2, NANOG) or membrane (SSEA4) staining intensity ≥2-fold above background (negative control: PBMCs).

### Assessment of genetic stability and prediction of the protein spatial structure of iPSCs

The successfully established iPSC cell line was compared with the genome of the patient’s blood (Figures 1A–C). The genetic stability of the *CLCNKB* gene was consistent between the constructed iPSCs and the blood, and no new mutations occurred. A mutation in c.228C > A in the *CLCNKB* gene was detected in this epilepsy family, which caused the codon of the compilation of the amino acid Cys to become a stop codon (p. Cys76Ter), resulting in early termination of peptide chain synthesis. The prediction of the protein spatial structure of the wild type versus the mutant protein is shown in Figure 1D.

### Differential gene analysis of three iPSCs

The RNA-seq differential gene analysis revealed a total of 1819 differentially expressed genes. By further applying a threshold of *p* < 0.05 and a fold change of 5, we identified 301 differentially expressed genes. Additionally, we clarify that both the father and son carry the same genotype related to the identified mutation. Comparative transcriptome analysis was performed between the patient (the son) and healthy (the mother) control, and also between the father and control. We found that the differentially expressed genes identified in the father were largely consistent with those found in the son. Nonetheless, to better represent the disease phenotype and for downstream functional analysis, we prioritized the patient (son)‘s data in the subsequent investigationFor both upregulated and downregulated genes, we selected the genes with the largest fold change for further analysis. Specifically, we chose the genes *TTTY14, TBL1Y, MEG8, MEG9, BCORP1,* and *RPAS4Y1* for analysis, all of which were found to be unrelated to neurological diseases. Additionally, by comparing the volcano plot of the disease group with that of the control group, we identified several significantly associated genes for analysis. The downregulated genes included *XIST, RAB17, PDGFB, MYOCD, and LARGE1.* These genes were selected because they met our predefined selection criteria and these genes were clearly visible in the volcano plot, indicating that they show strong differential expression between the compared groups. Notably, the clinical features of the patient involved uncontrolled hand movements accompanied by atrophy, and *MYOCD* and *LARGE1* may be related to this symptom. *MYOCD* is associated with muscular atrophy, whereas *LARGE1* is related to congenital muscular dystrophy. The upregulated genes included *NLRP2, RPSAP47, DDX3Y, TXLNGY,* and *MEG3,* which are unrelated to neurogenic diseases (Figures 1D,E).

## Discussion

In this study, we generated induced pluripotent stem cells (iPSCs) from a family affected by epilepsy. The family members included a phenotypically normal father and mother, and their son who exhibited epilepsy symptoms. Whole-genome sequencing of the patient’s family revealed a novel heterozygous nonsense mutation, c.228C > A, in the *CLCNKB* gene, where cytosine is substituted by adenine at nucleotide position 228. This mutation results in a premature stop codon, predicted to cause early termination of translation and produce a truncated protein. The truncated protein would lack essential functional domains, likely disrupting its normal function. *CLCNKB* encodes a voltage-gated chloride channel primarily expressed in the kidney, playing a crucial role in ion transport and electrolyte homeostasis. Hence, the structural disruption of this protein may significantly undermine its physiological function.

Since the *CLCNKB* mutation identified in this family has not been previously reported in relation to epilepsy, we aimed to explore the downstream transcriptional changes using transcriptome analysis. Instead of focusing solely on previously reported epilepsy-related genes, we extended our analysis to include both known and novel genes that may interact with *CLCNKB* and contribute to disease pathogenesis. This approach allows us to investigate potentially novel molecular mechanisms specific to this family’s genetic background. This novel mutation, not previously reported in the literature or found in public databases, is infrequent in the population and does not appear to be polymorphic. Notably, while the son’s genotype matches that of the father, their phenotypic expressions are markedly different, suggesting that other genetic or environmental factors may contribute to the disease phenotype. To further assess the impact of the mutation on the protein structure, we performed structural modeling using SWISS-MODEL for both the wild-type and mutated forms of the protein. As illustrated in Figure 1D, the mutation induces substantial conformational changes that may alter the protein’s interactions with regulatory molecules or its localization to the membrane.

Although we hypothesize that the mutation could potentially induce nonsense-mediated mRNA decay (NMD), this remains unverified experimentally and is a limitation of the current study. Our primary goal is to establish iPSC lines from this family to investigate the potential disease-causing genes associated with epilepsy and to conduct subsequent biological validation in future research. And this study identifies a novel heterozygous nonsense mutation in the *CLCNKB* gene, its precise biological impact and relevance to the disease phenotype remain to be elucidated through future functional studies. We performed RNA-seq on the constructed iPSCs and compared the data with those of normal human controls, identifying several significantly differentially expressed genes: *LARGE1, TBL1Y, TTTY14, MEG8*, and *MEG9*. We further analysed the correlation of these genes with neurodevelopment. Through in-depth investigation, we explored the possible pathogenic mechanisms of epilepsy in this family and the interactions between the genes involved.

iPSC models can be used for screening antiseizure drugs and studying their mechanism of action and targets. For example, the effects of different drugs, such as rapamycin and everolimus, on neuroinflammation and epilepsy can be compared by using the use of drugs (Li et al., 2024; Choi et al., 2023; Yang et al., 2017). iPSC-derived neurons can be used for the preliminary screening of possible antiseizure drugs as well as for exploring the mechanism of action of proven antiseizure drugs. For example, 4-aminopyridine (4-AP) treatment induces epileptiform activity in iPSC-derived neurons and is inhibited by standard antiepileptic drugs, supporting the first-line high-throughput screening of iPSC-derived human neurons to identify compounds with antiepileptic properties and rule out null compounds (Zhao et al., 2024; Shcheglovitov and Peterson, 2021).

Whole - genome sequencing of the patient’s peripheral blood samples identified several genetic variants in genes that, as per previous clinical reports, have been associated with disease conditions. However, the pathogenic significance of these variants still needs to be clarified, and their potential role in the observed phenotype requires further functional validation. Therefore, a detailed list of the identified variants is provided in the (Supplementary Table 1; Supplementary Figure 1), while specific gene functions are not elaborated on in the main text. RNA-seq analysis of the iPSCs revealed that genes such as *TTTY14, TBL1Y, MEG8, MEG9, BCORP1,* and *RPS4Y1* are not directly related to epilepsy. However, the downregulation of *MYOCD* and *LARGE1* may be associated with the patient’s muscle atrophy. Based on the patient’s clinical manifestations, previously reported disease - related genes, and our comprehensive RNA - seq data, we tentatively speculate that *CLCNKB* and *SLC12A1* may functionally coordinate in the pathophysiological context of epilepsy. RNA - seq analysis revealed that the expression of CLCNKB was upregulated in the patient when compared with control samples. This aberrant expression pattern may be correlated with alterations in other functionally - related genes, potentially contributing to the patient’s epileptic phenotype (Mcneill et al., 2020; Bilal Shamsi et al., 2021).

Furthermore, the potential roles of *CLCNKB* and *LARGE1* in epilepsy, especially in the context of ion channel dysfunction and the glycosylation modification, suggest that they may be key factors in the pathogenesis of epilepsy (Ma et al., 2024; Estévez et al., 2001). Although the direct effects of *CLCNKB* and *LARGE1* in epilepsy require further validation, their roles in ion channel function and the glycosylation modification offer new perspectives for research. These genes may be critical factors in the pathogenesis of epilepsy, particularly in the regulation of neuronal excitability and the stability of neural networks.

## Conclusion

In conclusion, our study suggests that the patient’s epilepsy may result from the combined effects of multiple genetic variations rather than a single gene mutation. We identified a novel, rare variant in the *CLCNKB* gene and found that other differentially expressed genes may be associated with the disease phenotype. Through RNA-seq analysis of iPSCs derived from the patient, we identified several significantly differentially expressed genes and analysed their potential correlation with neurodevelopment. We focused particularly on the roles of the *CLCNKB* and *LARGE1* genes, which are involved in regulating neuronal excitability and the glycosylation modifications, potentially implicating them in the pathogenesis of epilepsy. However, further validation through protein expression studies and in-depth functional analyses are needed to confirm these findings. Overall, the iPSC models generated in this study provide a valuable platform for investigating the complex genetic and molecular mechanisms underlying epilepsy and may contribute to the development of targeted therapeutic strategies.

## Statistical analysis

Graphpad Prism10.0 software was employed for statistical computation and analysis. Data measurements were represented as mean ± standard deviation. A *p* value of less than 0.05 signified a statistically significant difference.

## Data Availability

The datasets presented in this study can be found in online repositories. The names of the repository/repositories and accession number(s) can be found below: SAMN48836013 (http://www.ncbi.nlm.nih.gov/bioproject/1270669).

## Footnotes

^1^ http://geneontology.org/

## Supplementary material

SUPPLEMENTARY FIGURE 1 Interrelations among the *CLCNKB, PKN2, KIAA0586, FAT2*, and *COBL* genes. Analyzed the interactions among the identified genes and their upstream and downstream pathways using STRING (https://cn.string-db.org), thereby elucidating their relationships with the reported gene CLCNKB.

SUPPLEMENTARY TABLE 1 Gene names and their associated biological functions of the patient’s blood for sequencing to identify the relevant disease-causing genes.
