# Supplementary figures and images for: Development of patient-specific iPSC-based epilepsy models and identification of differentially expressed genes for disease mechanisms
Source: Front Neurosci. 2025 Jun 17;19:1582255. doi: 10.3389/fnins.2025.1582255 (PMC12209222; doi:10.3389/fnins.2025.1582255)

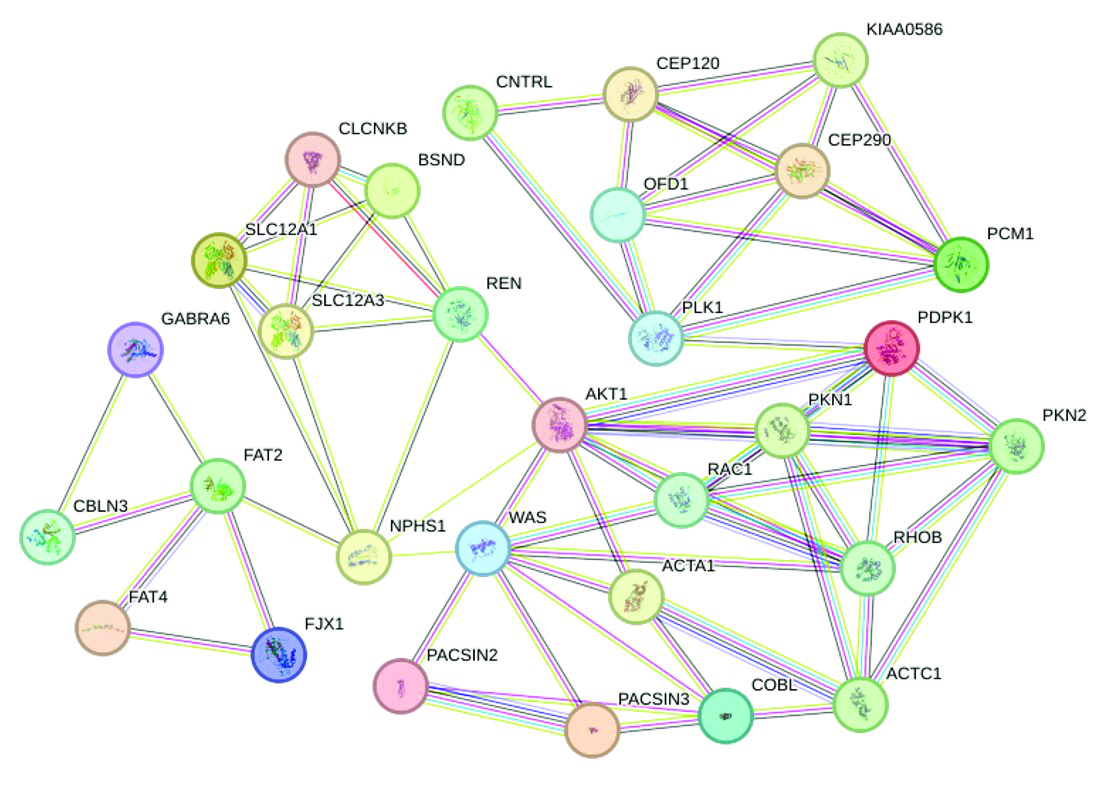

Supplement: SUPPLEMENTARY FIGURE 1 — Interrelations among the CLCNKB, PKN2, KIAA0586, FAT2, and COBL genes. Analyzed the interactions among the identified genes and their upstream and downstream pathways using STRING (https://cn.string-db.org), thereby elucidating their relationships with the reported gene CLCNKB. [file Image_1.tiff]
