# Supplementary material for: Development of patient-specific iPSC-based epilepsy models and identification of differentially expressed genes for disease mechanisms
Source: Front Neurosci. 2025 Jun 17;19:1582255. doi: 10.3389/fnins.2025.1582255 (PMC12209222; doi:10.3389/fnins.2025.1582255)
Supplement: SUPPLEMENTARY TABLE 1 — Gene names and their associated biological functions of the patient’s blood for sequencing to identify the relevant disease-causing genes. [file Table_1.docx]

S-Table 1：Gene Names and Their Associated Biological Functions

| Gene | Function |
| --- | --- |
| *CLCNKB* *(Chloride Voltage-Gated Channel Kb)* | CLCNKB encodes a voltage-gated chloride channel predominantly expressed in the kidney, where it mediates chloride conductance along nephron segments, including the thick ascending limb of Henle's loop, convoluted tubule, and the collecting duct. This channel plays a critical role in renal salt reabsorption and blood-pressure homeostasis. Mutations in CLCNKB are associated with autosomal recessive Bartter syndrome type 3, characterized by hypokalemic alkalosis with salt-wasting and low blood pressure |
| *PKN2* *(Protein Kinase N2)* | PKN2 is a serine/threonine-protein kinase related to protein kinase C and functions as an effector of Rho/Rac GTPases. It is involved in various cellular processes, including regulation of the cell cycle, actin cytoskeleton assembly, cell migration, adhesion, and transcription activation. PKN2 also plays roles in tumor cell invasion and apoptosis, and it alters key signaling pathways and transcriptional networks to regulate glucose and lipid metabolism |
| *KIAA0586* *(TALPID3)* | KIAA0586 encodes a conserved centrosomal protein essential for primary ciliogenesis and is involved in the Sonic hedgehog signaling pathway. It localizes to centrioles and is crucial for centrosome migration during ciliogenesis. Mutations in KIAA0586 are associated with Joubert syndrome, a ciliopathy characterized by cerebellar vermis hypoplasia and a distinctive "molar tooth sign" on brain imaging.​ |
| *FAT2* *(FAT Atypical Cadherin 2)* | FAT2 encodes a member of the cadherin superfamily, characterized by multiple cadherin-type repeats, EGF-like repeats, and a laminin G domain. It is involved in the regulation of cell migration, particularly in epidermal cells, and may modulate the organization of parallel fibers of cerebellar granule cells during development. FAT2 has been implicated in tumor invasion and is expressed in various normal and tumor tissues |
| *COBL* *(Cordon-Bleu WH2 Repeat Protein)* | COBL encodes a protein that plays a significant role in the reorganization of the actin cytoskeleton. It regulates neuron morphogenesis by increasing the branching of axons and dendrites, particularly in Purkinje cells. COBL binds to actin monomers and nucleates actin polymerization by assembling actin monomers in a specific orientation, promoting the growth of actin filaments at the barbed end |
